# Chemometric Characterization of Alembic and Industrial Sugar Cane Spirits from Cape Verde and Ceará, Brazil

**DOI:** 10.1155/2012/840528

**Published:** 2012-11-25

**Authors:** Regina F. R. Pereira, Carla B. Vidal, Ari C. A. de Lima, Diego Q. Melo, Allan N. S. Dantas, Gisele S. Lopes, Ronaldo F. do Nascimento, Clerton L. Gomes, Maria Nataniela da Silva

**Affiliations:** ^1^Departamento de Química Analítica e Físico-Química, Universidade Federal do Ceará, Campus do Pici, Fortaleza, Ce, Brazil; ^2^Departamento de Hidráulica e Engenharia Ambiental, Universidade Federal do Ceará, Campus do Pici, Fortaleza, Ce, Brazil; ^3^Instituto Federal de Educação, Ciência e Tecnologia (IFCE), Campus Acaraú, Ceará, Brazil

## Abstract

Sugar cane spirits are some of the most popular alcoholic beverages consumed in Cape Verde. The sugar cane spirit industry in Cape Verde is based mainly on archaic practices that operate without supervision and without efficient control of the production process. The objective of this work was to evaluate samples of industrial and alembic sugar cane spirits from Cape Verde and Ceará, Brazil using principal component analysis. Thirty-two samples of spirits were analyzed, twenty from regions of the islands of Cape Verde and twelve from Ceará, Brazil. Of the samples obtained from Ceará, Brazil seven are alembic and five are industrial spirits. The components analyzed in these studies included the following: volatile organic compounds (n-propanol, isobutanol, isoamylic, higher alcohols, alcoholic grade, acetaldehyde, acetic acid, acetate); copper; and sulfates.

## 1. Introduction

 Sugar cane spirit, or *cachaça,* is a typical Brazilian distilled beverage [1, 2]. This spirit is the second most consumed alcoholic beverage in the country and the third most consumed in the world. There are almost 30,000 *cachaça* producers in Brazil and over 5,000 *cachaça* brands available on the market [3].

 In Cape Verde, the sugar cane spirit, *grogue*, is a drink produced mainly by archaic practices, without supervision and without efficient control of the production process. To ensure the quality of *grogue*, it is necessary to correct deviations over the course of the whole production process. For Cape Verde, because there is still no local legislation, the results are evaluated according to Brazilian legislation.

 Mathematical and statistical methods of analysis can be used for diverse scientific purposes, such as selecting the measurements and procedures best suited to chemical experiments or obtaining a more accurate analysis of the resulting information. According to the needs of any particular study, chemometrics can be used for analytical signal processing, experimental planning and optimization, pattern recognition, data classification, multivariate calibration, and/or monitoring and modeling of various processes, among other applications [4–7]. 

 One of the first steps of chemometric analysis is to plot data in a multidimensional space, grouping the data with similar characteristics to demonstrate that there is some natural relationships between these data points. Thus, groups with distinct characteristics will be differentiated. 

 Exploratory multivariate analysis is performed in a matrix, and the data are organized in a spreadsheet where “*n*” samples with “*m*” variables results in a matrix [*n*  ×  *m*] [8, 9].

 Two methods were applied: the supervised method, which is applied when the classes of the samples are known and these data are used to build the model; and the unsupervised method, which is applied when there is no previous knowledge of sample classification and the samples are grouped according to the analysis of the acquired data.

 For pattern recognition, the chemometric method can be applied via hierarchical cluster analysis (HCA) or via principal component analysis (PCA). These techniques are based on the assumption that similar samples will be plotted closer together in multidimensional space than dissimilar samples [8–10]. 

 Principal component analysis was originally described by Pearson in 1901 and was subsequently applied by Hotelling in various scientific areas [8–10].

 Principal component analysis consists of redefining the coordinates of an axis system to make it more convenient for the analysis of a given set of samples. That is, *n* original variables generate, through their linear combinations, *n* principal components, which are obtained in decreasing order of maximum variance. Thus, the first principal component has more statistical information than the second principal component, which, in turn, has more statistical information than the third principal component, and so on. This method allows reduction of the dimensionality of the representative points of the samples [9, 11].

 The graph of principal component 1 versus principal component 2 provides window for the observation of data points in *n*-dimensional space. Principal component analysis can also be used to judge the importance of the original variables chosen. The original variables receive the highest weighting, or loading, in the linear combination of the first principal components and are thus the most important from a statistical point of view. The task of the professional who works with multivariate statistics is to interpret the point distributions on the graphs of the principal components and identify the original variables with the most weight in a linear combination of the most important principal components [11, 12].

 The technique of hierarchical clustering draws connections between the samples, producing a dendrogram in which similar samples are grouped together, and this similarity is a function of the distance between the points.

 The goal of this work was to develop analytical methods for the chemical characterization of sugar cane spirits and to use multivariate analysis to distinguish between the spirits according to their origin, quality, variety, and type.

## 2. Experimental

### 2.1. Instrumentation

A Shimadzu gas chromatograph (model GC-17A-FID) was used for the determination of volatile organic compounds present in samples of sugar cane spirits. The chromatographic column used was a DB-5 (30** **mx 0.25** **mm ID, 0.25** **mm thick film stationary phase of 5% phenyl and 95% dimethylsiloxane).

A Dionex ion chromatograph (ICS-3000) was used for the determination of anionic species. The chromatographic column used was an AS18 column (25** **cm × 4.0** **mm with an ASRS suppressor—Ultra II—4** **mm). Other components used included a conductivity detector, a 25**** *μ*L loop, and a potassium hydroxide (KOH) eluent generator.

Copper analysis was performed using a simultaneous inductively coupled plasma optical emission spectrometer with axial and radial views (Optima 4300 series, Perkin Elmer).

Acid digestions were performed in a digestion block (Tecnal, Piracicaba, SP, Brazil) equipped with Teflon vessels.

### 2.2. Reagents, Solutions, and Samples

All reagents used were of analytical grade, and the water (resistivity of 18.2** **MΩcm) was purified in a Milli-Q system (Millipore, Bedford, MA, USA).

The samples were collected from three islands of Cape Verde (Santiago, Santo Antão, and Brava) and also from Brazil (Ceará).  Table 1 presents the sample symbols used in this work and identifies the samples according to the region and the manufacturing process used.

Analytical grade solvents were used. Ethanol (VETEC, Rio de Janeiro, Brazil) had a purity of 99.5%. The analytical standards used, which were all 99.9% pure unless otherwise noted, were as follows: acetaldehyde (VETEC, Rio de Janeiro, Brazil); n-propanol (Merck, USA); ethyl acetate (Merck, USA); isobutanol (Merck, USA); butanol (Merck, USA) and isoamyl alcohol (Merck, USA).

Stock aqueous solutions of copper (1000 mg** **L^−1^) were purchased from Acros Organics (New Jersey, USA). The analytical calibration curve was prepared using 1.0, 5.0, 10, and 12 mg** **L^−1^ solutions of copper in 1.4 mol** **L^−1^ HNO_3_. 

### 2.3. Procedures

 For analysis of the volatile compounds, the oven's temperature program was as follows: an initial temperature of 40°C for 3 min which was increased at a rate of 5°C** **min^−1^ to 65°C, followed by a rate of 50°C** **min^−1^ to a final temperature of 200°C. The temperatures of the injector and the detector were 200°C and 300°C, respectively. Direct injection of samples was performed in split mode, with 1 mL of sample injected for gas chromatographic analysis.

 Sulfate analysis was performed using an elution program described as follows: 7.2 mmol between 0 and 10 min, increased to 22 mmol by 13.2 min, increased to 44 mmol by 13.44 min, and maintained at 44 mmol until the 15.0 min. The column temperature was 40°C. The current was 40 mA, and the conductivity was 0.6**** *μ*S.

 Copper was investigated after digestion of the samples. Aliquots of 2.0 mL sugar cane spirits and 2.0 mL HNO_3_ (68% w/w) were transferred to a Teflon vessel. The heating program of the digestion block was adjusted to increase the temperature to 120°C for 3 h. 

HCA was implemented in R using a stats package [14, 15] and was performed using a variation of Ward's linkage method [16], which adopted Euclidean distances as measures of dissimilarity. PCA was implemented using the FactoMineR (Factor analysis and data mining with R) package [17]. The approach used to implement PCA in FactoMineR is described in detail elsewhere [18]. In FactoMineR, the solution which maximizes the variance of the projected points is selected. Although rotational algorithms can help to improve the interpretability of the principal components, the FactoMineR method does not apply any rotation to keep its optimal property, the maximization of the variance of the projected points.

## 3. Results and Discussion

**Figure 1:**
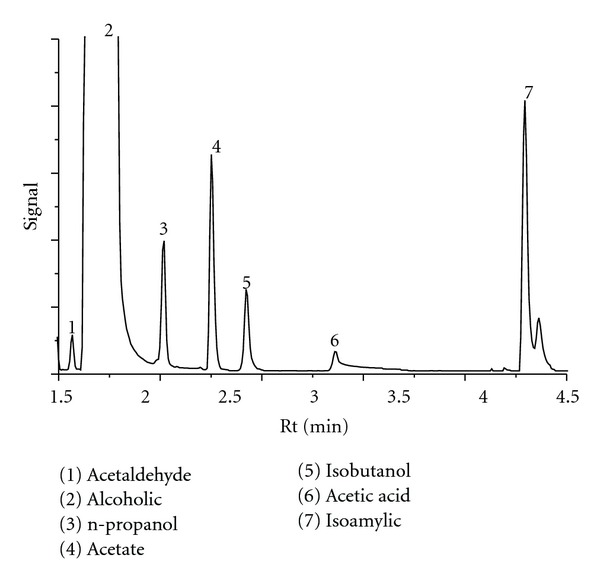
Chromatogram of a spirit from Cape Verde.

 The results of the chromatographic separation of the components present in the spirits from Cape Verde are shown in Figure 1. It was observed that total separation of the components occurred at a runtime of 4 min. Among the higher alcohols, the area of the isoamyl alcohol peak is remarkable not only for being the largest but also because it reveals a mixture of isomers (n-amyl and isoamyl alcohols).

 The concentrations of each component were evaluated according to Brazilian legislation using the values obtained from the chromatographic analysis. The results are shown in Table 2.

 The acetaldehyde content is an important parameter in controlling the processing quality of spirits. In Cape Verde spirits, the average acetaldehyde contents were 20 and 15.2 mg/100 mL AA for industrial and alembic spirits, respectively. For the Brazilian spirits, the average acetaldehyde contents were 21.8 and 11.9 mg/100 mL AA for the alembic and industrial, respectively (Table 4). Some spirit samples from Cape Verde (V3, V5, V7, V13, V19) and Brazil (B10 and B18) exceeded the limit of 30 mg/100 mL AA stipulated by Brazilian legislation.

 According to Almeida and Barreto, there is a correlation between the level of n-propanol and the quality of the spirits, such that the presence of higher levels of n-propanol occurs in lower-quality spirits [19]. In this work, the highest levels of n-propanol were found in the alembic spirits of Cape Verde. 

 Among the alcohols identified in Brazilian spirits, isoamyl alcohol is predominant, with average concentrations of 50.2 mg/100 mL AA and 120.3 mg/100** **mL AA for industrial and alembic spirits, respectively. In Cape Verde spirits, the averages were 100.2 mg/100 mL AA and 164.4 mg/100 mL AA for alembic and industrial spirits, respectively. 

 The sum total of the higher alcohol concentrations is low, with the exception of some Cape Verde samples (C8, C17, and C20), which exceeded the limit permitted by Brazilian legislation [20, 21].

 Spirits produced in copper stills are in high demand because of their unique taste [22]. However, the amount of copper in these spirits has been of concern as health problems can result when this element occurs in high concentrations [23]. The Ministry of Agriculture, Livestock and Supply (MAPA) has set a maximum limit of 5 mg L^−1^ for the distillation process [21].

 The copper concentrations of the samples show that more than fifty percent of the Cape Verde alembic spirits have levels above the maximum level of 5.0 mg L^−1^. Among the Brazilian spirits, only two samples exceeded the maximum level.

 One proposal to lower the copper content in the Cape Verde spirits is to replace the copper pot stills with stainless steel ones, thus eliminating the presence of copper salts available to be dissolved by the acid vapors during distillation and carried over to the final distillate [24].

 Other studies with Brazilian spirits have shown that the concentration of higher alcohols may vary depending on the region (Table 3). 

 Nascimento et al. studied the components of spirits from Sao Paulo and obtained concentrations of 135.62 and 187.42 mg/100 mL AA in copper pot stills and stainless steel stills, respectively. In this work an average of 235.2 mg/100** **mL AA for alembic spirits from Ceará (Table 3) was obtained [13, 25].

**Figure 2:**
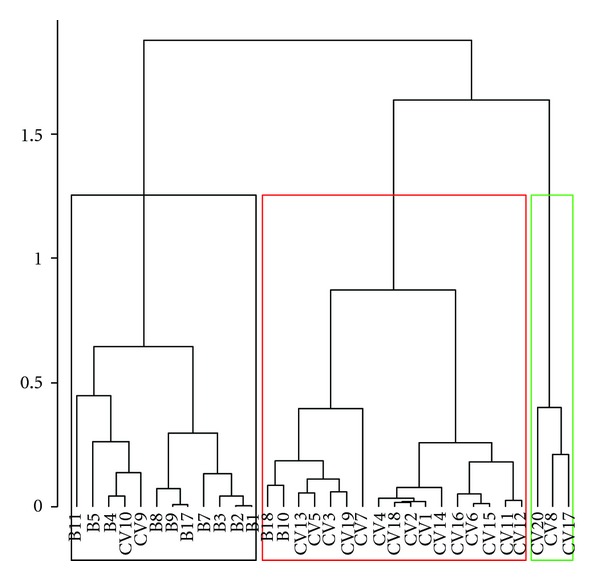
Sugar cane spirits dendrogram.

Figure 2 shows the dendrogram of sugar cane spirits based on the chosen variables. It was possible to observe the formation of four main groups that separated the Brazilian sugar cane spirits from those of Cape Verde. It was noted that alembic sugar cane spirits from Cape Verde, as well as some Brazilian ones, were concentrated more in the first group (first branch) and that the Brazilian industrial spirits have a distinct profile from that of Cape Verde industrial spirits.

 According to Muñoz et al., the vectors farthest from zero correspond to the variables with the greatest influence on the principal component value, while vectors closer to zero indicate variables with little influence on the principal component [26].

In this study, it was revealed that the variables with the greatest influence on the principal components 1 and 2 were sulfates and the higher-level alcohols, isobutanol and isoamylic alcohol (Table 4).

**Figure 3:**
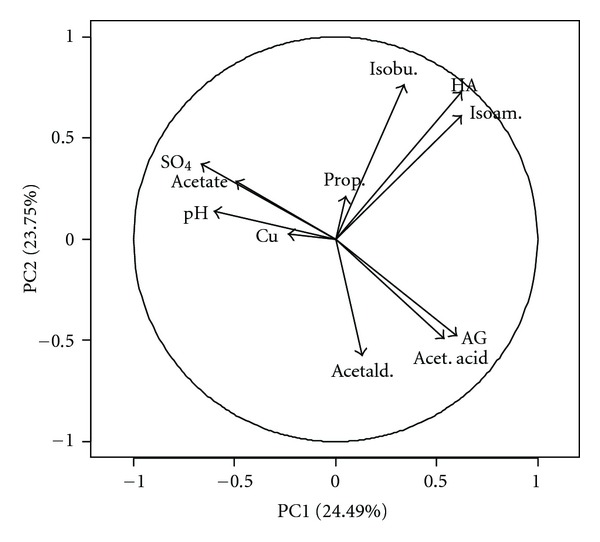
Loadings plot.

 In the loadings plot (Figure 3), the principal components 1 and 2 contain negative and positive values. Principal component 1 shows that higher-level alcohols, isobutanol and isoamylic alcohol, were present in high levels in the sugar cane spirits from Cape Verde when compared to those from Brazil. Both principal components 1 and 2 presented high levels of higher alcohols in sugar cane spirits from Brazil.

**Figure 4:**
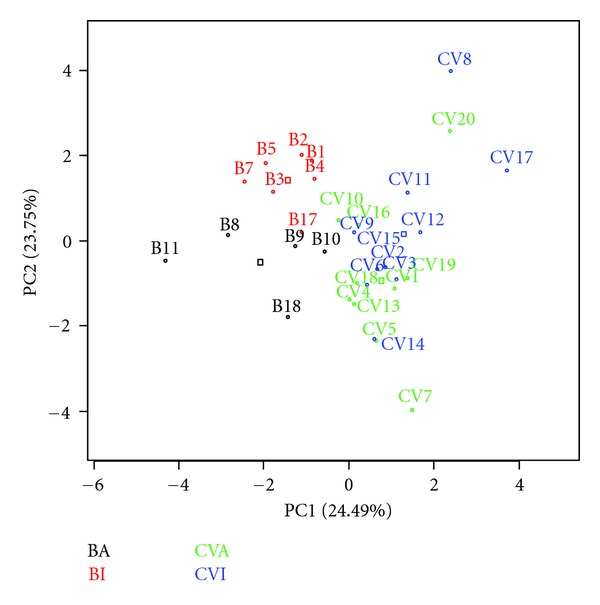
Graph of “scores.”

 In the graph of the “scores” (Figure 4), it is possible to observe that principal component 1 separates Brazilian (B) sugar cane spirits from those originating in Cape Verde (V) and discriminates between Brazilian industry-produced and alembic spirits. These observed subgroups are due to differences in quality control, and it was observed that industrial spirits from Brazil have a very narrow profile while industrial spirits from Cape Verde exhibit higher dispersion.

 PCA is a useful technique for finding patterns in high-dimensional data and for plotting those data in a way that highlights the similarities and differences among the points. The first component accounts for most of the variability in the data, and each succeeding component accounts for the highest variance possible under the constraint that it is orthogonal to (i.e., uncorrelated with) the preceding components [27].

In PCA, the data matrix is decomposed into scores and loading matrices. The scores vectors describe the relationship among the samples in the model subspace, and the loadings vectors describe the importance of each descriptor within the model.

 For this work, principal component 1 describes 24.49% of the original information and principal component 2 describes 23.75%. The cumulative percentage of principal components 1 and 2 was 48.24%. Four principal components were required to describe 70.78% of the original data.

## 4. Conclusions

 Chemometric analysis enabled the extraction of more information for data analysis, promoting interpretation of the results with greater security and reliability.

 The results showed that both hierarchical cluster and principal component analysis were able to separate sugar cane spirits from Cape Verde from Brazilian sugar cane spirits, thus highlighting the differences in production of sugar cane spirits between the two countries. 

 For alembic and industrial samples, PCA analysis indicated that sugar cane spirits from Cape Verde are disparate, while sugar cane spirits from Brazil are more closely grouped, indicative of the greater control over sugar cane spirit manufacturing in Brazil.

## Figures and Tables

**Table 1:** Identification of the spirits of Cape Verde and Brazil.

Cape Verde	Symbol
Santiago	V1—Alembic
Santiago	V2—Industrial
S. Antão	V3—Industrial
Santiago	V4—Alembic
Santiago	V5—Alembic
Santiago	V6—Industrial
Brava	V7—Alembic
Santiago	V8—Industrial
Santiago	V9—Industrial
Santiago	V10—Alembic
Santiago	V11—Industrial
S. Antão	V12—Industrial
Santiago	V13—Alembic
Santiago	V14—Industrial
Santiago	V15—Industrial
Santiago	V16—Alembic
Santiago	V17—Industrial
S. Antão	V18—Alembic
Santiago	V19—Alembic
Santiago	V20—Alembic
Ceará	B1—Industrial
Ceará	B2—Industrial
Ceará	B3—Industrial
Ceará	B4—Industrial
Ceará	B5—Industrial
Ceará	B7—Industrial
Ceará	B8—Alembic
Ceará	B9—Alembic
Ceará	B10—Alembic
Ceará	B11—Alembic
Ceará	B17—Industrial
Ceará	B18—Alembic

**Table 2:** Concentrations (mg/100 mL AA*) of compounds in Cape Verde and Brazilian spirits.

Variable	BA	BI	CVA	CVI
n-propanol	60.9 ± 19.9	65.4 ± 37.8	78.3 ± 59.2	64.5 ± 41.9
Isobutanol	27.02 ± 8.9	46.5 ± 9.5	33.1 ± 15.1	41.1 ± 22.4
Isoamylic	50.18 ± 40.5	120.3 ± 23.0	100.2 ± 49.4	164.4 ± 94.7
Higher alcohols	156.9 ± 62.6	235.2 ± 42.3	222.3 ± 89.6	282.6 ± 104.7
Alcoholic grade	39.8 ± 7.1	38.8 ± 0.4	45.4 ± 3.6	44.9 ± 2.7
pH	4.79 ± 1.2	4.3 ± 0.3	3.8 ± 0.4	3.9 ± 0.3
Acetaldehyde	21.8 ± 13.3	11.9 ± 5.7	20.0 ± 15.3	15.2 ± 7.8
Acetic acid	17.1 ± 13.4	17.5 ± 13.7	67.7 ± 68.7	72.0 ± 45.6
Acetate	1.2 ± 0.8	1.5 ± 1.1	0.5 ± 0.3	0.6 ± 0.5

^∗^AA: Absolute alcohol; BA: Brazilian alembic; BI: Brazilian industrial spirits; CVA: Cape Verde alembic; CVI: Cape Verde industrial spirits.

**Table 3:** Values of the volatile components found in different Brazilian spirits.

Spirits/region	Components	Values (mg/100 mL AA)	Reference
Minas Gerais; Brazil	Acetic acid	81.93	[7]
	Higher alcohols	179.13	
	Acetate	61.19	
	Acetaldehyde	13.56	

São Paulo; Brazil (copper)	Acetic acid	2.07	[13]
	Higher alcohols	135.65	
	Acetate	1.63	
	Acetaldehyde	1.9	

São Paulo; Brazil (steel)	Acetic acid	2.15	[13]
	Higher alcohols	187.42	
	Acetate	10.8	
	Acetaldehyde	0.9	

CE; Brazil (steel)	Acetic acid	17.5	This work
	Higher alcohols	235.2	
	Acetate	11.9	
	Acetaldehyde	1.5	

**Table 4:** Eigenvalues and cumulative variances of the first five principal components.

Variables	PC1	PC2	PC3	PC4
Alcoholic grade	0.6044	−0.4789	0.1078	−0.0229
pH	−0.6046	0.1420	0.1327	−0.0762
Acetaldehyde	0.1308	0.5791	0.4502	0.3579
n-propanol	0.0499	0.2163	0.7784	−0.4609
Acetic acid	0.5419	−0.4954	0.0932	0.4859
Isobutanol	0.3407	0.7691	0.2454	0.1995
Isoamylic	0.6277	0.6165	−0.2626	0.2052
Higher alcohols	0.6257	0.7344	0.1686	−0.0009
Sulfate	−0.6685	0.3758	−0.0599	0.3816
Acetate	−0.4949	0.2872	0.0876	0.4114
Copper	−0.2363	0.0275	0.6527	0.2403
